# High Cable Forces Deteriorate Pinch Force Control in Voluntary-Closing Body-Powered Prostheses

**DOI:** 10.1371/journal.pone.0169996

**Published:** 2017-01-18

**Authors:** Mona Hichert, David A. Abbink, Peter J. Kyberd, Dick H. Plettenburg

**Affiliations:** 1 Delft Institute of Prosthetics and Orthotics, Department of Biomechanical Engineering, Delft University of Technology, Delft, The Netherlands; 2 Delft Haptics Lab, Department of Biomechanical Engineering, Delft University of Technology, Delft, The Netherlands; 3 Institute of Biomedical Engineering University of New Brunswick, Fredericton, Canada; 4 Department of Engineering Science, University of Greenwich, Chatham Maritime, United Kingdom; Semmelweis Egyetem, HUNGARY

## Abstract

**Background:**

It is generally asserted that reliable and intuitive control of upper-limb prostheses requires adequate feedback of prosthetic finger positions and pinch forces applied to objects. Body-powered prostheses (BPPs) provide the user with direct proprioceptive feedback. Currently available BPPs often require high cable operation forces, which complicates control of the forces at the terminal device. The aim of this study is to quantify the influence of high cable forces on object manipulation with voluntary-closing prostheses.

**Method:**

Able-bodied male subjects were fitted with a bypass-prosthesis with low and high cable force settings for the prehensor. Subjects were requested to grasp and transfer a collapsible object as fast as they could without dropping or breaking it. The object had a low and a high breaking force setting.

**Results:**

Subjects conducted significantly more successful manipulations with the low cable force setting, both for the low (33% more) and high (50%) object’s breaking force. The time to complete the task was not different between settings during successful manipulation trials.

**Conclusion:**

High cable forces lead to reduced pinch force control during object manipulation. This implies that low cable operation forces should be a key design requirement for voluntary-closing BPPs.

## Introduction

### Myo-electric prostheses

It is generally asserted that upper-limb prosthesis operation requires sufficient feedback to obtain adequate dexterous manipulation [1,2]. Myo-electric prostheses require visual confirmation of movements of the terminal device as there is no other direct form of feedback about the action of the prehensor. Several approaches to pinch force feedback have been investigated in the last decades such as vibro-tactile feedback [3,4], mechano-tactile feedback (pressure on skin) [5–7], electro-tactile feedback (electro-cutaneous stimulation) [8–10], skin stretch [11], and force feedback spanning the joint [12]. None of them have been implemented in commercial myo-electric prostheses and all except the latter target tactile feedback. However, in dynamic force feedback tasks, proprioception is the key player and tactile feedback has only an ancillary role [13].

### Body-powered prostheses

The first body-powered prosthesis (BPP) was designed by Ballif in 1818 [14]. Current BPPs still rely on the same principle: A shoulder harness captures the relative motion of shoulder and arm movements and transmits their action via a Bowden cable to operate a prosthetic prehensor. Two types of prehensors are used: Voluntary-Closing (VC) and Voluntary-Opening (VO) which open or close when the cable is pulled. The VC BPP provides the user with Extended Physiological Proprioception (EPP) [15]. EPP extends the concept of proprioception to tools connected to the body, in this case a prosthesis. This has the inherent benefit of direct proprioceptive feedback about the prehensor’s movement and forces through the movement and forces of the harness.

To date, body-powered hooks are equally preferred to myo-electric hands [16]. Stated advantages of body-powered prostheses compared to myo-electric prostheses [17–19] include mass, robustness and cost-efficiency. However, BPPs are still far from optimal in spite of the advances since the patenting of the Dorrance split hook in 1912. Body-powered hands are less preferred than hooks [16]. A user might prefer a prosthetic hand instead of a hook for cosmetic reasons, but then he needs to exert 1.5–8 times more mechanical work and will experience 2–27 times higher hysteresis or energy dissipation [20]. Further advances in harness design [16], reduction of friction in the transmission [20,21], and weight reduction of the prosthesis [19] are possible. Fundamental improvement in BPP design could be realized by optimizing the relationship between the forces and displacements at the prehensor and those at the shoulder harness [22]. Progress is currently impeded by the limited understanding of how cable forces influence grasping performance and comfort.

### Cable forces in prosthesis operation

Current BPPs usually require high operating forces [22], which lead to pain and fatigue during or after operation [16] and may additionally disturb the feedback and control of pinch forces. Previous work in our group demonstrated that the control of operation forces decreases with higher cable forces [23].However, these experiments were done without prehensor and objects. This means the dynamic effects of prosthesis–object interaction and compensatory strategies of the user were not considered. Therefore, the effect of high cable operation forces for prosthesis-object-interaction remains unexplored.

This study aims to quantify the influence of high cable forces on the accuracy of pinch force control, when a VC BPP is used to grasp an object and transport it without exceeding pre-defined force boundaries. We hypothesize that high cable operation forces reduce the task performance in terms of the amount of successfully transported objects.

### Approach

Able-bodied subjects were equipped with a by-pass socket and BPP. They were instructed to execute a repeatable abstract task of grasping an object and transferring it to another predefined position. The object transfer involves arm movements, which influence the pinch forces if the subject does not correct for this effect. Therefore the object transfer simulates the type of challenges that VC body-powered prosthetic users experience in daily activities. In order to inherently include interaction force limits in this manipulation task, a “mechanical egg” [5] was used which offers repeatable limits: at too little force subjects can’t lift it, and at an adjustable level it “breaks” mechanically. Abstract collapsible objects have been used in diverse studies investigating feedback and pinch force control [5,24], since they offer a natural challenging dynamic grasping task. As prosthetic users aim to execute grasping tasks as quickly as they would with an intact hand, time to execute the task was taken as an outcome measure. Subjects were asked to execute the task as quickly as they could without breaking the object. Breaking an object in daily life is inconvenient and is generally avoided.

## Materials and Methods

### Subjects

Twelve able-bodied male subjects (11 right & 1 left handed, age: 30±8 (mean±SD) years old, height: 179±5 cm weight: 88±8 kg) participated in this study. The data of one of the subjects was excluded from data analysis because he was unable to successfully complete 80% of the trials. In addition, the force data of a second subject were not available for analysis. None of the subjects had experience operating BPPs. The Research Ethics Board of the University of New Brunswick, where the experiments were conducted, approved the experiments (REB #2014–064). All subjects signed an informed consent form prior to the experiments.

### Apparatus

**Fig 1:**
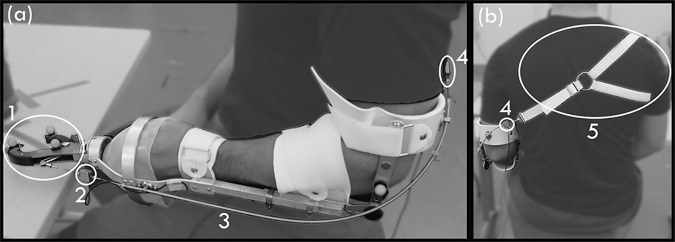
Apparatus. Side-view (a) and back-view (b) of one subject wearing the custom-made bypass-prosthesis. The prehensor (1) is connected to the fitting. The prehensor (1) was connected via a Bowden cable (3) to the “figure-of-nine” harness (5). The Bowden cable forces were measured before and after the outer cable housing with force sensor 1 (2) and force sensor 2 (4).

Subjects were fitted with a custom-made prosthesis consisting of a modified prehensor, which was attached to an adjustable bypass fitting, and linked to an adjustable “figure-of-nine” harness to provide the cable forces to close the prehensor (Fig 1). The equipment was manufactured and modified in the Atlantic Clinic for Upper Limb Prosthetics in Fredericton, Canada. The length of the socket was adapted to the subject’s lower arm. Likewise, the harness could be modified and adapted to the subject’s shoulder width and upper arm length. A standard 1/16” (.159 cm) diameter stainless steel cable (C100) running through a cable housing for C-100HD cable (CH-100HD). To reduce friction in the cable a Teflon liner for heavy duty cable housing (CH100-HD) (all from Hosmer Dorrance Corporation, Chattanooga, USA) was placed in the inside of the cable housing. The coefficient of friction was reported to be 0.092 and assuming a maximum cable curvature of 90 degrees we would expect the static efficiency of force transmission of the Bowden cable to be more than 90% according to Carlson et al. [25].

#### Prehensor

The voluntary-closing Grip 3 prehensor (TRS Inc., Boulder, USA) was chosen because of its mechanical efficiency and linear relationships between cable operation forces and cable excursions as well as between cable operation forces and pinch forces (Fig 6 and 10 in [20]). The relationship between the pinch force and the cable force of a non-deformable object was determined to be

$$\frac{F_{pinch}}{F_{cable}}=0.64$$ (1)

**Fig 2:**
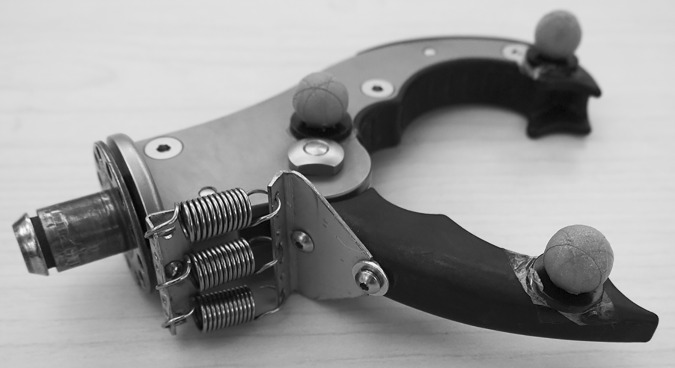
Prehensor. TRS hook with the internal torsion spring replaced by external linear springs in the high force setting (3 x 1.7 N/mm springs); 2 x 0.11 N/mm springs were used for the low force setting.

The cable force required to start building up a pinch force is dependent on the prehensor’s spring stiffness and the prehensor’s opening. Thus, with small modifications, the prehensor could facilitate different cable force settings to generate the same pinch force. The original prehensor’s torsion spring was replaced by interchangeable linear springs of different stiffness fixed at the prehensor’s thumb lever (Fig 2). The settings consisted of either two parallel springs (0.11 N/mm each), or three parallel springs (1.7 N/mm each). These different settings then required either low or high cable forces to close the prehensor. The high force setting (~40–50 N) represents the required forces to operate a TRS hook, a Hosmer APRL hand or hook as shown in the study of Smit and Plettenburg (Fig 10 in [20]). The low force setting (~10–15 N) was chosen according to the preferred forces of prostheses users as shown in the results of a preliminary study of our group [23].

#### Test object: “mechanical egg”

**Fig 3:**
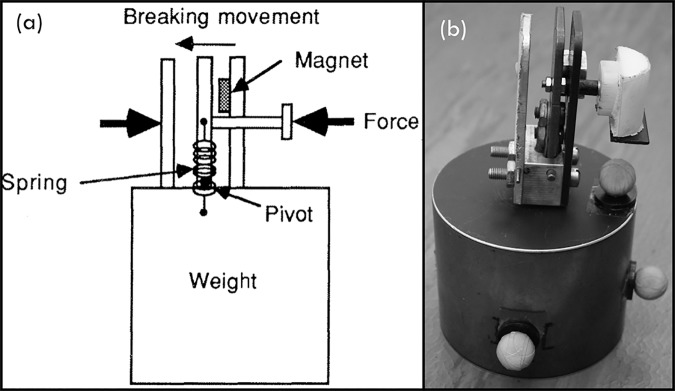
Test object. The “mechanical egg’s” breaking mechanism [5] is shown in the left picture (a) and the experimental setup is shown to the right (b).

Subjects needed to interact with a force-sensitive test object (Fig 3). The object was called a “mechanical egg” since it “breaks” when excessive pinch force is applied. This “mechanical egg” is the same device as designed and used in the study of Meek et al. [5]. The original grasping surface of the egg was rounded to match the TRS finger’s shape and covered with non-slip material (Dycem Ltd, Bristol, UK) at the finger and the thumb grasping surface, in order to enhance the grip quality. The weight of the object (and thereby the slipping force) remained constant during the experiments (385 g).

**Fig 4:**
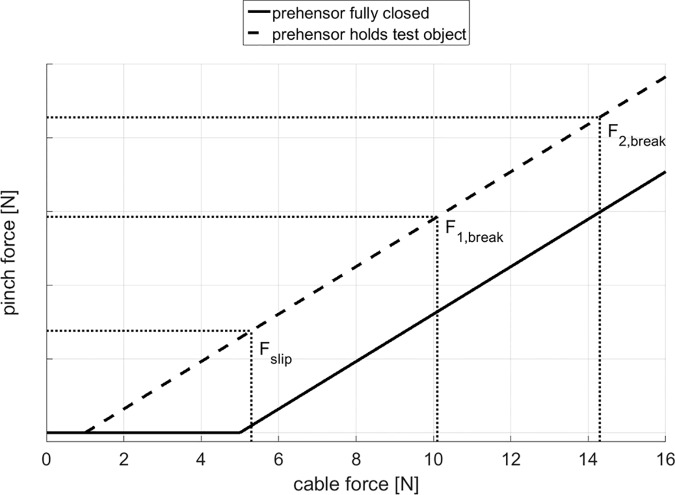
Cable to pinch force. The cable force to pinch force relationship is shown when the TRS hook is fully closed and when the test object is held utilizing the prehensor’s low spring stiffness setting. The force at which the object slips out of the prehensor (F_slip_), and the forces at which the “mechanical egg” breaks span the operating window in which the test object can be manipulated, for both the low (F_1,break_) and high (F_2,break_) breaking force settings. Note that the cable force at which the TRS hook starts to build up a pinch force on the test object is an estimation, since it was not experimentally determined. As a consequence the pinch force values are not representative.

The object’s breaking force was adjusted to a high and low breaking force setting, resulting in two pinch force margins at which the egg will not break or drop during manipulation. Table 1 contains the statically determined cable forces for both prehensor’s spring stiffness settings at which the object slips of the prehensor (F_slip_), thus the minimum required cable force to hold the test object, and the cable forces at which the object breaks for the low and high breaking forces (F_break_). Fig 4 illustrates the relationship between cable and pinch forces. For training purposes, a third setting with an even higher breaking force was applied.

**Table 1:** The statically determined minimum required cable forces to hold the “mechanical egg” (F_slip_) and its maximum allowed cable forces (F_break_) for the two object’s breaking force settings derived for the prehensor’s two spring stiffnesses.

spring stiffness	0.22 N/mm	0.22 N/mm	5.1 N/mm	5.1 N/mm
breaking force	high	low	high	low
**minimum required cable force (F**_**slip**_**) [N]**	5.3±0.3		28.8±0.3	
**maximum allowed cable force (F**_**break**_**) [N]**	14.3±1.3	10.1±0.8	42.2±0.6	38.8±0.4

#### Measured signals

A custom-made timer was pressed by the subject to indicate the start and end of each trial. The subject reported the task completion time to the experimenter. Cable operation forces were measured at both the forearm and back of the subject. Forces were measured with two mini S beam 222N load cells (FUTEK Advanced Sensor Technology, Inc., Irvine, United States), amplified with a CPS amplifier (SCAIME S.A.S., Juvigny, France) and fed into the analogue input of a motion capture system (Vicon Motion Systems Ltd., Oxford, United Kingdom) at 1000 Hz. The signals were recorded using Nexus 1.8.3 software (Vicon Motion Systems Ltd., Oxford, UK), and stored for off-line analysis after each trial. The recorded motion capture data were not used for the current study.

### Metrics

The number of failures and the time required completing the task served as the task metrics. Prosthetic users should be able to manipulate objects efficiently without breaking or dropping them.

### Procedure

**Fig 5:**
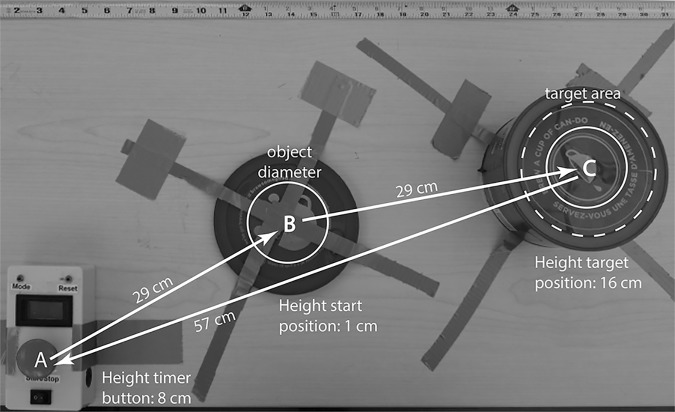
Visualization of one trial. The subject hits the self-timer button A to start the time measurement, moves 29 cm to grasp the object at the lower area B, then moves the object 29 cm to the higher target area C. After releasing the object, the subject needs to hit the self-timer to stop the time measurement.

Each subject wore the bypass-prosthesis on the left arm (Fig 1) and was seated at a table (height: 73cm). After adjusting the prosthesis and the seat to a height comfortable for each subject, the training session commenced. Subjects were instructed to operate the prosthesis using shoulder protraction of the right side, and humeral adduction and anteflexion of the left side and had freedom of choice in their control movements. First, the subject familiarized themselves with the operation of the device by moving wooden blocks (2.5 x 2.5 x 2.5 cm) from the predefined low (1 cm above the table) to high position (16 cm above the table), start position B to target position C in Fig 5. Training continued with the “mechanical egg”, starting with the stiffest setting, followed by the two test conditions, the high and low breaking force settings. Once the subject was familiar with the “mechanical egg’s” function at the training setting, the timer (A in Fig 5) was introduced. For training purposes, each setting had to be conducted at least 10 times with 3 successful trials in a row before subjects moved on to a lower breaking force setting. Training ended when they could successfully execute the trial at the egg’s low force setting.

The four experimental conditions were tested in a counterbalanced order, combinations of low and high cable forces and low and high breaking force setting. A trial consisted of starting the timer with the prosthesis, transferring the test object from the low to high position, and stopping the timer. The subjects were instructed to transfer the egg as quickly as possible without breaking or dropping it (Fig 5). Subsequently, the subject reported the time or a failure to the experimenter. Each of the four experimental conditions was tested 25 times, resulting in a total of 100 trials per subject. After the experiment was completed, the subjects were asked during a semi-structured interview which system they preferred, the low or the high cable force setting, and why they preferred that system.

### Data analysis

For 11 subjects the number of failures and the average times over the 25 trials per condition were analyzed with a repeated measures ANOVA (IBM SPSS Statistics Version 20—IBM Corporation, Armonk, United States).

The recorded Voltage of the force sensors was converted into Newton and filtered with a 3^rd^ order filter (filtfilt function) at 10 Hz (Matlab Version 2013b—The MathWorks, Inc., Natick, United States) for 10 subjects. The peak forces (maxima) were determined for each successful trial and averaged per condition.

Friction losses were determined by comparing measured input and output forces of the Bowden cable.

## Results

The prehensor’s high spring stiffness of 5.1 N/mm resulted in a 3.5 to 4 times higher cable operation force measured at the forearm than the low prehensor’s spring stiffness (0.22 N/mm) as indicated in Table 2.

**Table 2:** The peak forces for successful trials measured at the forearm (2 in Fig 1) and at the back (4 in Fig 1) of the subject and averaged over all subjects per condition.

spring stiffness	0.22 N/mm	0.22 N/mm	5.1 N/mm	5.1 N/mm
breaking force	high	low	high	low
**force@forearm (F1) [N] (mean±std)**	12.6±0.9	10.7±0.9	43.5±2.1	42.0±2.5
**force@back (F2) [N] (mean±std)**	15.5±1.2	13.3±1.2	51.5±2.2	49.8±2.9
**efficiencies Bowden cable**	81%	80%	84%	84%

**Fig 6:**
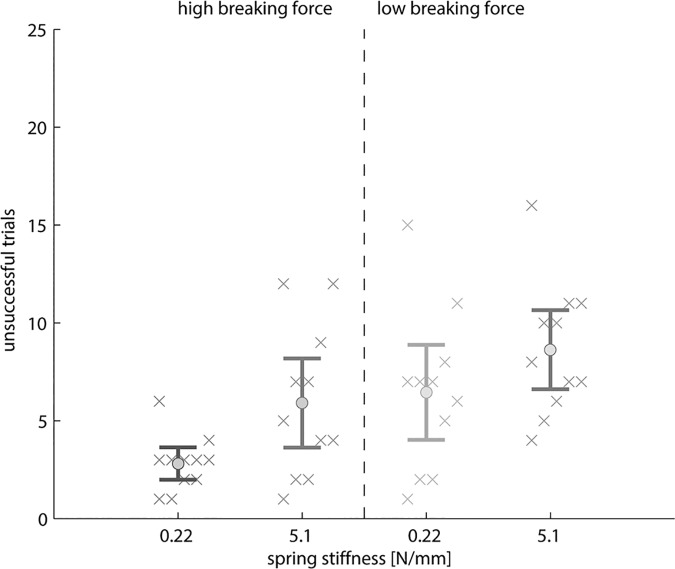
Number of unsuccessful trials. The number of unsuccessful trials out of 25 trials per condition are indicated by “x” per subject (N = 11), averaged over all subjects (“o”) with the 95% confidence intervals (whiskers). The results are compared for the high (left) versus the low (right) breaking force setting of the test object as well as the low (0.22 N/mm) versus high (5.1 N/mm) spring stiffness of the prehensor.

**Fig 7:**
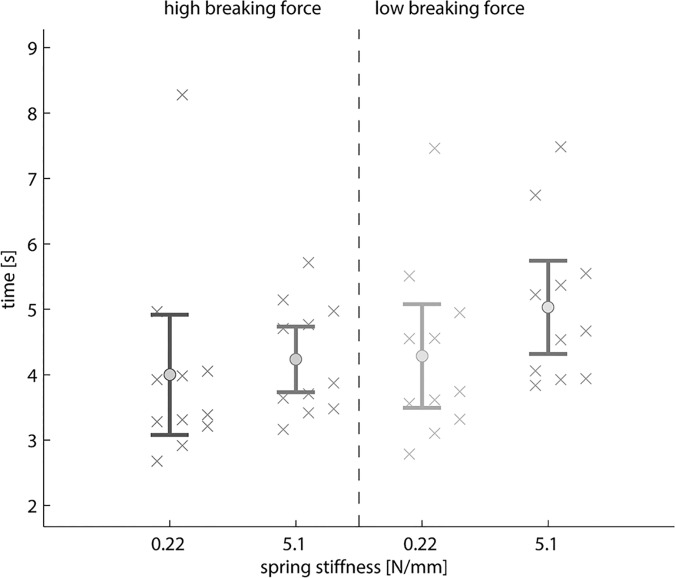
Task completion time. The time to complete the experimental task was determined by the average of all successful trials per condition per subject (N = 11) indicated by “x”. The error bars represent the average of all subjects (“o“) with the 95% confidence intervals (whiskers). High (left) versus low (right)) breaking force setting of the test object as well as the low (0.22 N/mm) versus high (5.1 N/mm) spring stiffness of the prehensor were compared.

High cable operation forces resulted in significantly more unsuccessful trials (F(10,1) = 6.763, p = 0.026, Fig 6). The task completion time, however, was not significantly affected by the magnitude of the cable force (F(10,1) = 4.097, p = 0.071, Fig 7).

Subjects exerted significantly less force on the control cable during the task execution at the object’s low breaking versus the high breaking force condition (Table 2; force@forearm: F(9,1) = 114.608, p<0.001; force@back: F(9,1) = 123.013, p<0.001). The low object’s breaking force resulted in significantly more unsuccessful trials than the high breaking force setting (F(10,1) = 25.817, p<0.001, Fig 6). Subjects completed the experimental task significantly quicker at the high object’s breaking force setting (F(10,1) = 25.346, p<0.001, Fig 7).

The prehensor’s spring stiffnesses and the object’s breaking forces did not show interaction effects for the number of unsuccessful trials (F(10,1) = 0.225, p = 0.646) and the average task execution time (F(10,1) = 1.461, p = 0.255).

The outcome of the semi-structured interviews of the subjects showed that ten of the eleven subjects preferred the low spring-stiffness setting. The reported reasons for the low spring-stiffness system preference were the ease to control and distinguish pinch force and a higher long term comfort (less load on the axillar region, less tiring, less required effort).

## Discussion

This study aimed to quantify the influence of high cable forces on the accuracy of pinch force control, when a VC BPP is used to grasp an object and transport it, while not exceeding pre-defined force boundaries. To create two different cable force levels, springs with different endpoint stiffnesses were mounted on the prehensor. We hypothesized that high cable operation forces reduce the task performance. The results showed that higher cable operation forces resulted in more unsuccessful trials and thus an inferior task performance compared to lower cable forces, which is in line with our hypothesis. Subjective interview reports showed that subjects preferred the lower cable forces as well. Interestingly, high cable forces did not increase the task execution time. This finding might indicate that the subjects either did prioritize the task execution time over the successful task performance, or were not aware of their accuracy in controlling the decreased pinch force. A recent study has shown that high cable operation forces result in a larger deviation in the targeted cable forces [23]. The TRS prehensor cable forces relate linearly to the pinch forces as shown in Fig 10 of [20]. Consequently, a wider deviation of pinch forces could be expected at higher cable forces. This wider deviation seems to result in decreased pinch force control accuracy. Required cable forces for a 15 N pinch force range from 33 (TRS hook) to 131 N (Hosmer soft hand) for VC prehensors [20]. The cable forces of this study ranged from 16 to 52 N (as measured at the subjects’ back) for the two cable force conditions and show effects in pinch force control accuracy. This emphasizes the urgency of lowering the required cable operation forces for VC BPPs to achieve better pinch force control. Additionally, users of body-powered hands also complained about “slowness in movement, insufficient grip strength and high-energy expenditure” [17]. These problems can be tackled with lower cable operation forces.

The difficulty of the task was manipulated by utilizing two breaking force settings of a “mechanical egg”. The narrower the object’s grasping force margin, the more critical the pinch forces on the object became. Thus the task gets more challenging, which is indicated by the higher number of failures and the longer task execution time in the low breaking force setting. Fragile objects were hypothesized to require more attention from the user and the manipulation to be more time-consuming than rigid objects. We did not find interaction effects between the magnitude of breaking and cable force. Thus irrespective of the task difficulty, we can conclude that higher cable operation forces deteriorate the pinch force control accuracy.

The differences of cable forces measured simultaneously at the forearm and at the back of each subject are striking (Table 2). These differences are mainly due to friction, a well-known disadvantage of Bowden cables, which increases with the curvature of the cable [25]. In our experiment the friction losses result in efficiencies between 80 and 84% of the exerted forces, despite the Teflon liner in the outer cable housing to improve the efficiency of force transmission. According to Carlson, in a static set up an efficiency of 80% implies a cable curvature of approximately 150 degrees [25]. Since the angle was never more than 90 degrees in this set up, it would suggest that there is a different behavior of the Bowden cable during dynamic prosthesis operation. This corresponds to recent evidence presented at the ISPO Europe conference 2016: Preliminary results on the dynamic properties of different types of Bowden cables were discussed [26], that suggest decreasing efficiencies for increasing cable velocities. Unfortunately, in this experiment cable velocities were not measured, preventing further analysis on the impact of dynamic properties of the Bowden cable on the pinch force control accuracy and the human controller’s abilities to anticipate for this apparently unknown behavior of the system. However, it is clear that the Bowden cable introduces additional inefficiencies above those of the prehensor [20,21]. Interestingly, the measured cable forces during the object transfer task at the prehensor’s 5.1 N/mm spring stiffness and object’s low breaking force (Table 2) exceed the statically determined cable forces at which the “mechanical egg’ breaks (Table 1). We speculate that dynamic effects might have allowed subjects to exceed the statically determined breaking forces.

### Study limitations

Instead of amputees, the experiment was performed by able-bodied subjects without experience in prosthesis operation. The task performance might differ between subjects with and without arm deficiencies due to different anatomy and the lack of experience in prosthesis operation. Experienced users might have developed strategies to grasp several objects efficiently in activities of daily living (ADL). However, a predefined, non-varying grasping surface was utilized in this experiment, removing the need to develop different grasping strategies. The subjects learned quickly how to grasp the test object due to the intuitive operation of a VC prehensor and were already able to distinguish grasping forces during the training session. In a previous study [23], no differences in the deviations of the controlled forces were found between subjects with and without arm deficiencies. As a consequence, performance differences between prosthetic users and our healthy control subjects are not expected for these experimental conditions.

The prosthesis simulator was placed at the left arm, which was the non-dominant arm of 10 out of the 11 subjects. This is presumed to best reflect the actual situation of prosthesis users, who usually prefer to manipulate objects with their natural hand, making the affected side the non-dominant side. The effect of operating the prosthesis simulator with the dominant versus the non-dominant side was not investigated.

The task instructions of transferring the “mechanical egg” as quickly as possible from and to a predefined position without breaking the “egg” might have been interpreted in different ways. Subjects might have prioritized the task execution time over the number of failures or vice versa. If a penalty for failure would have been applied, the subjects would probably all have prioritized to execute the task successfully rather than completing the task as quickly as possible. This might also explain why we did not find significant effects in the task execution time for the two cable force settings.

### Implications and future research

Design requirements for BPPs lack quantitative values especially when considering the user’s capacities and demands. This study clearly shows that pinch force control should be improved when utilizing low cable operation forces in the VC prosthetic design. However, there might be a disadvantage to reducing cable operation forces in terms of perception. A preliminary study showed that cable operation forces between 20–30 N are the preferred forces with fixed cable excursions [23]. Plettenburg et al. suggest a relationship between the strength of the user and the preferred operation forces. This relationship as well as the influence of cable excursions on the preferred forces need to be investigated. Additionally, the optimal ratio between cable operation forces and pinch forces is yet to be determined. Future research should prioritize achieving a satisfactory grip strength at the best possible sensory feedback as a design criterion [16].

A second crucial factor for the BPPs efficiency is the reduction of friction. The unpredictable behavior and the inefficiencies of the Bowden cable during dynamical task execution suggest a need for better solutions in BPPs design. The effects of the Bowden cable on pinch force control accuracy are unclear. This impedes the development of better prostheses.

Another crucial factor for BPPs is the harness design: The primary concern for users is skin irritation, pain and exhaustion during or after operation of a BPP [16,17]. The results of the semi-structured interviews imply a higher long term comfort (less load on the axillar region, less tiring, less required effort) with lower cable operation forces. However, users feel restricted in their movements due to the harness. New harness designs are required. One commercially available alternative to the harness is the ipsilateral scapular cutaneous anchor [27]; a patch is glued to the back of the user and is connected to the Bowden cable. The ability to distinguish operation forces compared to the traditional harness and the range of possible operation forces is unclear. Additionally, with a new harness design the appearance as well as the ease of donning and doffing the prostheses could be improved, which are two additional user design preferences [17].

The study of Lum et al. shows that fragile object manipulation is inferior with a prosthesis than with the intact biological hand [24]. However, the performance of the VC BPP user was exceptional compared to the other prosthetic users. This single user performed the task without breaking a fragile object as successful as the able-bodied controls. Although it was a single user, it emphasizes the high potential benefits users may gain with improved BPPs design. This study indicated the benefits of one of the BPPs design criteria: a decreased cable operation force. More BPPs design criteria should be quantified, like the required pinch forces to manipulate objects and the resulting transmission ratio between cable forces and pinch forces for optimal VC BPP operation.

## Conclusion

The goal of this research was to quantify the influence of high cable forces on object manipulation with a voluntary-closing, body-powered prosthesis. Lower cable operation forces lead to better control as shown by fewer unsuccessful trials, even though lower cable forces had no effect on task execution time. For the experimental conditions studied, we conclude that a lower cable force leads to improved performance during object manipulation. Therefore, we argue that low cable operation forces should be a key design requirement for voluntary-closing BPPs.

## Abbreviations

BPP: Body-powered prosthesis
VC: voluntary-closing
VO: voluntary-opening
